# P241: Nosocomial infections in very low birthweight infants in germany: current data from the national surveillance system neo-kiss

**DOI:** 10.1186/2047-2994-2-S1-P241

**Published:** 2013-06-20

**Authors:** R Leistner, B Piening, P Gastmeier, C Geffers, F Schwab

**Affiliations:** 1 Institute of Hygiene and Environmental Medicine, Charité Berlin, Berlin, Germany

## Introduction

Infants with very low birthweight (<1,500 g, VLBW) are at increased risk for nosocomial infections (NI). In the year 2000, we implemented a surveillance system for VLBW infants in Germany: NEO-KISS. In 2005, a joint committee of healthcare providers and insurance companies required German neonatology departments to participate. As a result, NEO-KISS is now a nationwide surveillance system for NI in VLBW infants.

## Objectives

We present NEO-KISS data collected between 2007 and 2011 by 228 neonatology departments.

## Methods

Rates of sepsis, pneumonia and necrotising enterocolitis (NEC) were calculated. In order to evaluate the department-specific infection rate we introduced a new indicator: the Standardised Infection Rate (SIR). The SIR considers the department-specific patient distribution (based on the patients’ birthweight) and describes the ratio of observed and expected infections (calculated from the reference data for this individual patient distribution).

## Results

The data presented comprise 33,048 VLBW infants. The overall incidence density of sepsis was 4.7 per 1,000 patient days and of CVC-associated sepsis 8.6 per 1,000 CVC-days. The incidence of pneumonia was 0.6 per 1,000 patient-days and of pneumonia among mechanically ventilated patients 2.7 per 1,000 ventilator days. The incidence of NEC was 0.8. The median number of VLBW infants per department in 2010 was 30 (IQR 12-49).

## Conclusion

The SIR showed strong variation among the participating departments. It is an excellent tool for identifying outliers in nosocomial infection rates and for stimulating activities to decrease the risk of nosocomial infections.

## Disclosure of interest

None declared

